# Reducing Research Waste Through Good Reporting Practices

**DOI:** 10.2188/jea.JE20160105

**Published:** 2016-08-05

**Authors:** Yasuyuki Okumura

**Affiliations:** Research Department, Institute for Health Economics and Policy, Association for Health Economics Research and Social Insurance and Welfare, Tokyo, Japan

**Keywords:** reporting guidelines, STROBE, STREGA

Roughly 85% of healthcare research funding may be wasted due to several avoidable reasons, including poor research question selection, poor study design, selective non-publication, and poor reporting.^1^ The cost of waste worldwide was estimated at 200 billion United States dollars in 2010.^1^ All actors in the research field—researchers, institutions, regulators, funders, publishers, and policy makers—have important roles in waste reduction.^1^ To reduce waste from poor reporting, many high-impact medical journals endorse and actively implement reporting guidelines that specify a minimum set of items required for a clear and transparent account of what was done and what was found in study.^2^ Over 300 reporting guidelines have so far been published for specific types of research.^3^ Key reporting guidelines include the CONSORT statement for randomized controlled trials, the STROBE statement for observational studies, the STARD statement for diagnostic accuracy studies, and the PRISMA statement for systematic reviews.

In this issue, Nedovic and colleagues report a systematic review evaluating the effectiveness of journal endorsement of the STREGA statement, which is an extension of the STROBE statement for genetic association studies.^4^ The authors identified 18 journals in the field of genetics and heredity that endorsed STREGA in their instructions for authors. They compared articles published before and after the year of endorsement for adherence to STREGA. Their results showed a 14 percentage point improvement in overall adherence, derived from the 22 items of STREGA (63% vs 49%). Specifically, large improvements were observed for “Study size,” “Variables,” “Quantitative variables,” and “Data sources/measurement.” The authors also identified impact-factor-matched journals that have never endorsed STREGA. Their results showed an 8% higher adherence in journals endorsing the STREGA compared to those that have never done so (63% vs 56%) and no significant improvement in overall adherence in journals that have never endorsed STREGA. The authors concluded that STREGA should be endorsed in the journals’ instructions for authors in the field of genetics and heredity.

In general, journals follow two primary strategies to endorse and implement reporting guidelines. The first is a requirement (or recommendation) in the journals’ instructions for authors, as in the *Journal of Epidemiology*, that reporting guidelines should be followed. The second is an editorial requirement that authors submit a reporting guidelines checklist together with their manuscript. Although journal endorsement of reporting guidelines might improve poor reporting, adherence to reporting guidelines remains sub-optimal. Indeed, Nedovic et al showed adherence of <50% for 8 of 22 items even among journals that have endorsed the STREGA statement; therefore, the authors recommend the use of the latter strategy.^4^

However, I believe that both journal endorsement strategies have a limited effect on adherence to reporting guidelines. Cobo et al conducted a randomized controlled trial evaluating the efficacy of an additional review based on reporting guidelines.^5^ Compared with journal endorsement, they used a much more intensive strategy, in which a statistician provided suggestions on how to follow reporting guidelines during peer review. Their results showed that an additional review based on the reporting guideline improved adherence but that the observed effect was smaller than expected. One potential explanation for this is that some researchers may not have understood the details of reporting guidelines when they were conducting their research, which might make it difficult for them to improve their manuscripts during peer review. Cobo et al suggest that researchers should anticipate future requirements of adhering to reporting guidelines at the very beginning of their research.^5^

To improve poor reporting, it is necessary to provide more opportunities to researchers and reviewers (and even editors) to learn reporting guidelines. The Enhancing the QUAlity and Transparency Of health Research (EQUATOR) network has provided several such opportunities.^3^ For example, a 3-hour workshop was held on September 7, 2013 in Chicago for editors and reviewers to successfully launch an editorial policy for reporting guidelines. In addition, a 5-day workshop was held in Oxford on July 6–11, 2015 for researchers to effectively write, publish, and disseminate research. Some educational materials are available on the EQUATOR website.^3^ Researchers, reviewers, and editors will benefit from participating in such workshops, which might contribute to waste reduction in research. Similar efforts should be made in entire scientific societies to provide learning opportunities for reporting guidelines.
